# Ag-fiber/graphene hybrid electrodes for highly flexible and transparent optoelectronic devices

**DOI:** 10.1038/s41598-020-62056-1

**Published:** 2020-03-20

**Authors:** Yooji Hwang, Junhee Choi, Ji-Weon Kim, Jin-Woo Lee, Jae Geun Kim, Ha Hwang, Kwang Wook Choi, Wanghoon Lee, Byeong-Kwon Ju

**Affiliations:** 1 Display and Nanosystem Laboratory, School of Electrical Engineering, Korea University, 145, Anam-ro, Seoul, 02841 Republic of Korea; 2 Department of Micro/Nano Systems, Korea University, Seoul, 02841 Republic of Korea; 3 R&D Team, Haesung DS Co., Ltd. 508, Teheran-ro, Gangnam-gu, Seoul, 06178 Republic of Korea; 4 Realistic Media Research Centre, Gumi Electronics and Information Technology Research Institute, 202, 3D Display Centre 350-27, Gumidaero, Gumi, Gyeongbuk 39253 Korea

**Keywords:** Mechanical and structural properties and devices, Mechanical and structural properties and devices, Nanowires, Nanowires

## Abstract

Transparent conducting electrodes (TCEs) have attracted considerable attention towards the development of flexible optoelectronic devices. In this study, mixed-dimensional TCEs are fabricated based on the two-dimensional graphene and one-dimensional electrospun metal fiber that can address the shortcomings of each electrode. In comparison with other TCEs, the Ag fiber/graphene hybrid electrodes exhibited a highly stable morphology (67% lower peak-to-valley ratio), low sheet resistance (approximately 11 Ω/sq), high transmittance (approximately 94%), high oxidation stability with excellent flexibility, and outstanding chemical stability. The multiple functionalities of the transparent and flexible hybrid structure highlight its potential for applications in emerging electronics and highly stable optoelectronics.

## Introduction

Recently, as the demand for wearable devices increases, optoelectronic devices, such as, liquid-crystal displays (LCD)^1^, organic light-emitting devices (OLEDs)^2,3^ and organic solar cells (OSCs)^4^, have also been developed in more flexible forms. As interest in these flexible optoelectronic devices increases, researches on TCEs for implementing such devices are being actively conducted. Conventional indium tin oxide (ITO) electrode has been applied in optoelectronic devices owing to high transmittance and conductivity^5^. However, the ITO electrode has fatal shortcomings, and its strain values are significantly low (approximately 1.1%), rendering it unsuitable for use as an electrode for emerging optoelectronic devices. Additionally, it has a high refractive index, and it requires a high vacuum and high-temperature deposition which makes ITO unsuitable for roll-to-roll manufacturing^6^. In this regard, various alternatives, such as carbon nanotubes (CNTs)^7^, graphene^8,9^, metal nanowires^10,11^, metal nanogrids^12^, and thin films^13^, that can replace ITO have been investigated in TCEs.

Among these alternatives, one-dimensional (1D) metal networks, such as metal nanowires and metal fiber electrodes, have been studied extensively as promising substitutes for ITO. Numerous studies on metal networks have been conducted and their applications^14,15^ have been stimulated owing to their excellent electrical properties (10 Ω/sq), high optical transmittance (approximately 90%), and high strain value (approximately 11%), compared to ITO. Furthermore, these 1D metal networks do not require a high vacuum and high-temperature deposition process, which could be implemented in a roll-to-roll manufacturing^16^. Among the metal-network–based electrodes, the electrospun Ag fiber has attracted considerable interest because of its lower percolation threshold, as a result of its longer length (a few cm), compared to that of Ag nanowires (AgNWs), and thus has been applied in various optoelectronic devices^15,17^. Moreover, the surface roughness of the junction-free electrospun Ag fiber can be simply controlled by modifying the thickness of its Ag thin-film deposition, which is a critical problem for AgNWs.

However, because Ag fiber electrodes are also made of metal, their electrical characteristics are deteriorated by heat and chemical treatments when applied in device fabrication. Furthermore, owing to the nature of the metal when exposed to air and moisture, the Ag fiber electrodes are oxidized easily, which leads to a significant increase in resistance and haziness. Therefore, an additional buffer layer is required that can prevent the oxidation of the Ag fiber electrodes while maintaining their electrical characteristics. Additionally, because the Ag fiber electrodes are fabricated in a network form, current only flows through the Ag fiber, and no current flows into the empty space between the Ag fiber electrodes^18^. Therefore, an OLED with Ag fiber electrodes exhibits non-uniform emissions at a low luminance because the current only flows through the fiber electrodes^19^. Thus, the fabrication of an ultrathin, gas- and thermal-stable, conductive, and flexible buffer layer for the Ag fiber electrodes is required, which can improve the non-uniform current flow caused by the network structure while maintaining the durability of the Ag fiber electrodes.

In recent studies, hybrid structures of graphene and metal nanowires have been considered alternatives to existing electrodes such as ITO for emerging electronics^20,21^. Graphene, a two-dimensional (2D) graphite structure, is considered the most promising material with outstanding electrical, optical, and thermal properties^22,23^. Particularly, graphene has excellent mechanical strength as it has surprisingly high strain values (approximately 25%). Furthermore, graphene has its unique honeycomb structure as a result of its single atomic carbon layer, which prevents small molecules such as hydrogen and water from passing through and keeps it stable against chemical agents^24^. Therefore, several studies have used graphene as a protection layer to prevent the oxidation of metals such as copper and nickel^25^. Additionally, graphene exhibits absorption of only 2.3% in the NIR range to visible light wavelengths, which minimizes the loss of transparency from the Ag fiber electrodes^25,26^.

In this study, highly stable and flexible mixed-dimensional TCEs were fabricated by wet-transferring graphene manufactured by the chemical vapor deposition (CVD) method onto junction-free Ag fiber electrodes for applications in optoelectronic devices. The new nanomaterial derived from the 1D Ag fiber and 2D graphene features excellent electrode properties by complementing each other’s disadvantages while highlighting their advantages. By raising the graphene buffer layer on top of the Ag fiber electrodes, additional current flowed through the graphene, and the percolation threshold was decreased accordingly, thereby improving the overall current characteristics. Furthermore, graphene’s properties markedly improved the thermal and chemical durability of the electrodes. These Ag-fiber/graphene hybrid electrodes are expected to be applied to emerging optoelectronic devices that can feature excellent characteristics while replacing the existing electrodes.

## Results and Discussion

### Fabrication of graphene-based TCEs

**Figure 1:**
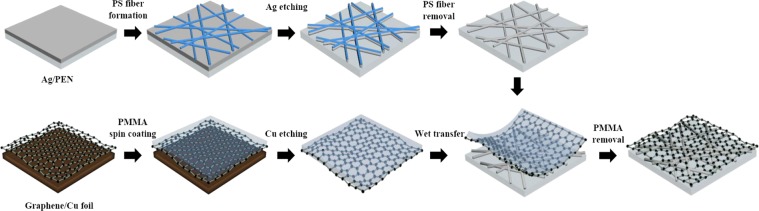
Schematic of the fabrication of hybrid electrodes.

Figure 1a presents a schematic of the fabrication process for the Ag-fiber/graphene hybrid electrodes; the electrodes were fabricated by wet-etching the Ag thin film, which was not covered by a polystyrene (PS) fiber mask, and subsequently by simply wet-transferring the CVD graphene on the Ag fiber electrodes (see Methods).

**Figure 2:**
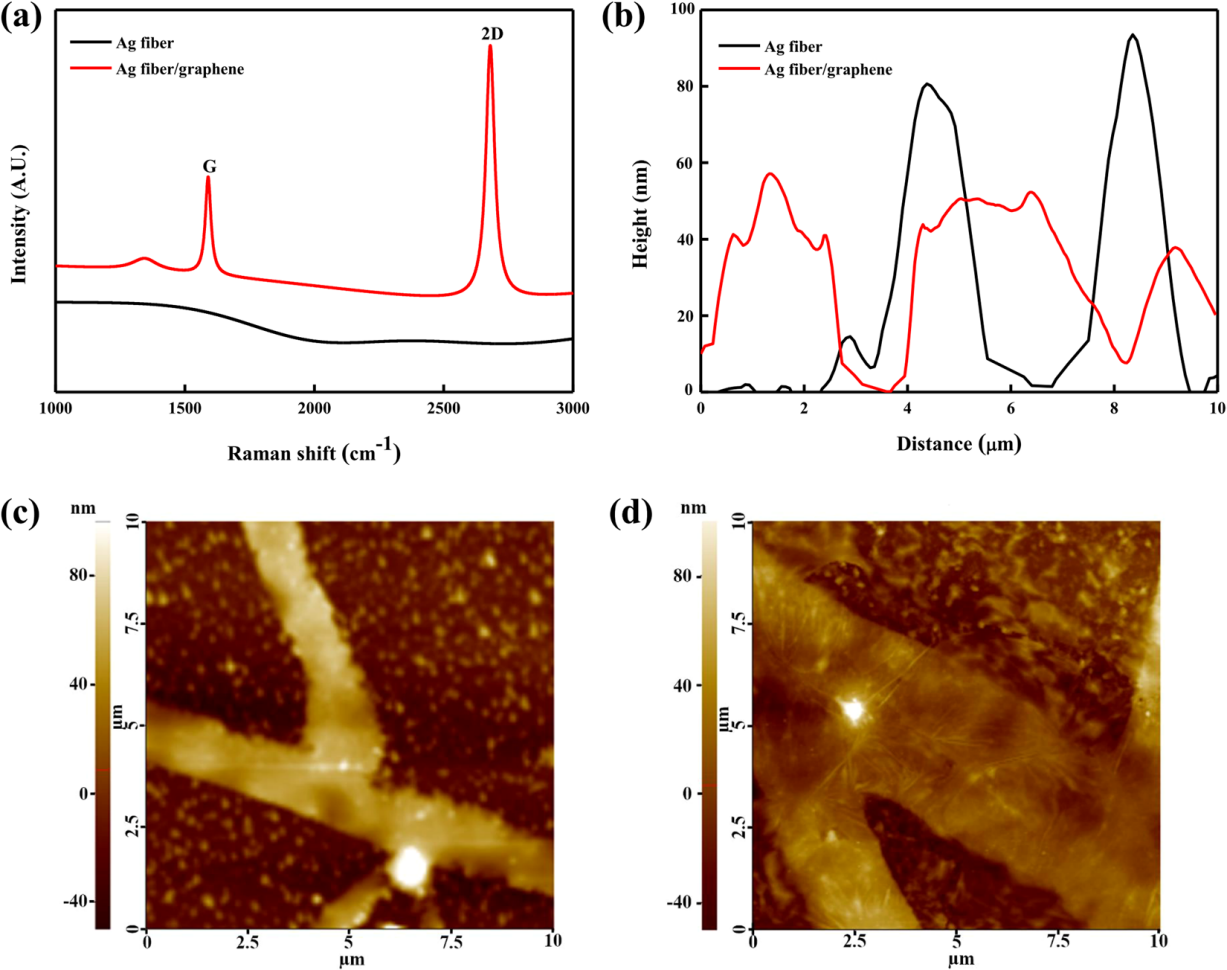
(**a**) Raman spectra and (**b**) line profiles of the Ag-fiber/graphene and Ag fiber; Comparison between their morphologies using AFM: (**c**) Ag fiber and (**d**) Ag-fiber/graphene on the PEN substrates.

A Raman spectrum was acquired directly from the Ag-fiber/graphene hybrid electrodes to confirm the presence of graphene on the hybrid electrodes. Figure 2a shows two main peaks in the Raman spectra of CVD monolayer graphene: G peak, common to graphite-related materials at 1,580 cm^−1^ and 2D peak, showing the number of layers of graphene at 2,700 cm^−1^. Here the 2D-to-G intensity ratio was larger than 1 and there was no D band, showing the presence of disordered structure, around 1,350 cm^−1^. This result confirms that the wet-transfer of CVD monolayer graphene onto the Ag fiber electrodes was successful without any damage^27^.

Morphological images of the Ag fiber and Ag-fiber/graphene hybrid electrodes were acquired by using AFM. A lower surface roughness enables strong bonding and easier post-processing, and it improves the device performance^28^. High peak-to-valley ratio can cause electrical shorts and current leakage, which can degrade the performance of the device. 1D metal networks are not suitable for many device applications owing to their intrinsic roughness, and this causes a high leakage of currents, which leads to severe device failures^29,30^. Hence, even if the surface roughness is lower than that of AgNW, the highly porous surface morphology of the single Ag fiber is not appropriate for applications in thin-film devices. In this regard, the surface roughness of the Ag fiber can be controlled by simply modifying the thickness of its Ag thin-film deposition. A Ag thickness of 40 nm was used to fabricate the Ag fiber electrodes. Figure 2b presents the AFM line profiles, whereas Fig. 2c,d present the AFM images of the Ag fiber and Ag-fiber/graphene hybrid electrodes on the polyethylene naphthalate (PEN) substrates, respectively. The height of the Ag fiber electrodes was approximately 90 nm, whereas the height of the Ag-fiber/graphene hybrid electrodes was approximately 50 nm, which is 67% smaller than that of the pristine Ag fiber. The encapsulation of the Ag fiber with the graphene monolayer significantly decreased the root-mean-square (RMS) surface roughness (*R*_rms_) with the larger width of the Ag fiber electrodes. The average RMS surface roughnesses and average peak-to-valley ratio for the Ag fiber and Ag-fiber/graphene electrodes are summarized in Table 1. The *R*_rms_ values of the Ag fiber and Ag-fiber/graphene electrodes were 32.719 and 15.676, respectively. These results demonstrate that the Ag-fiber/graphene hybrid electrodes are suitable for applications in emerging thin-film optoelectronic devices.

**Table 1:** Roughness of the Ag-fiber/graphene electrodes.

	*R*_rms_ (RMS average)	*R*_a_ (roughness average)
Ag fiber	32.719	30.188
Ag-fiber/graphene	15.676	11.372

### Optical and electrical characteristics of the Ag-fiber/graphene hybrid electrodes

**Figure 3:**
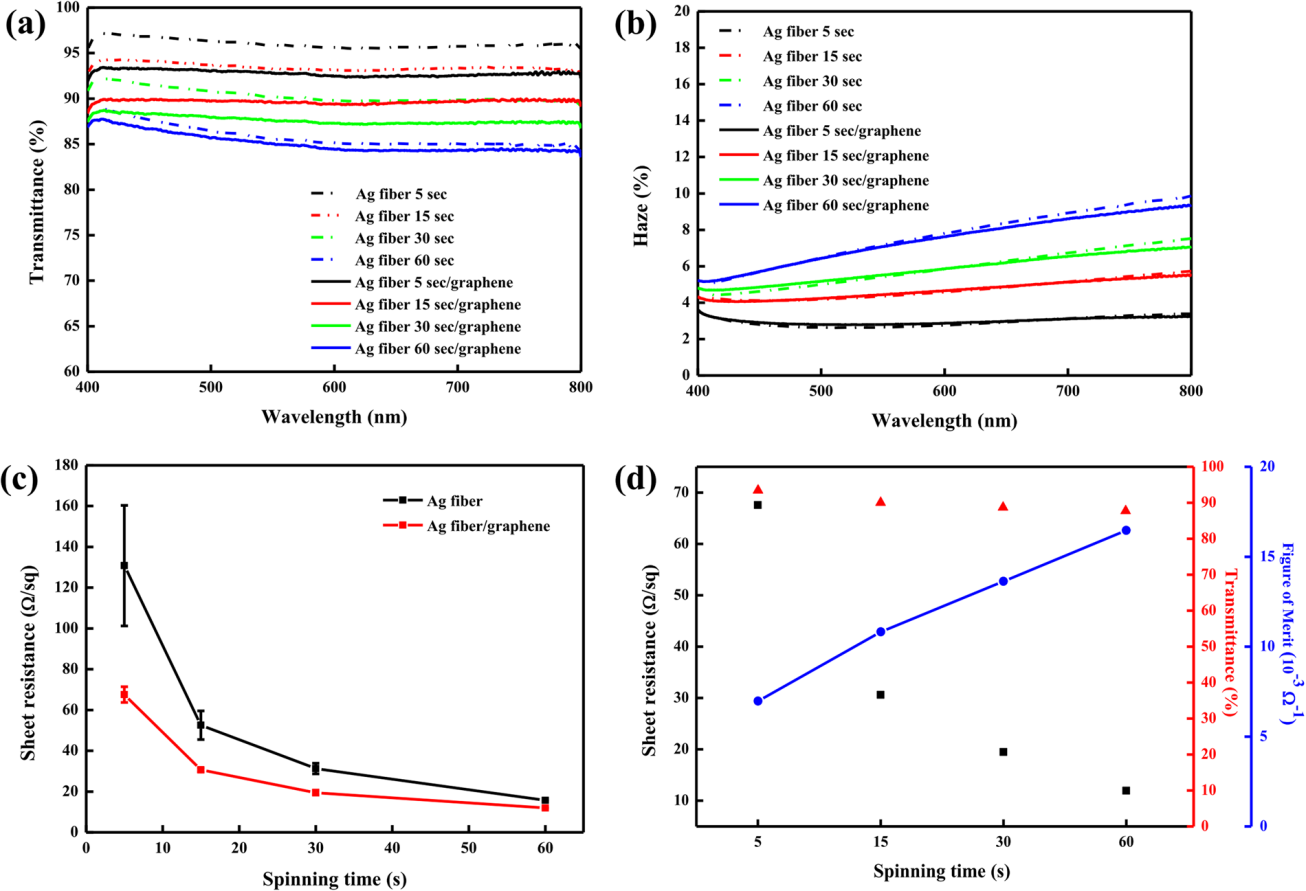
(**a**) Total transmittance, (**b**) haze, and (**c**) sheet resistance of the Ag fiber and Ag-fiber/graphene hybrid electrodes obtained by using different spinning times of 5, 15, 30, and 60 s; (d) FoM for Ag-fiber/graphene hybrid electrodes as a function of spinning time.

The optical and electrical properties of the Ag fiber and Ag-fiber/graphene hybrid electrodes were investigated. First of all, Fig. 3a describes the transmittance of the hybrid electrodes as a function of the presence of transferred graphene monolayer and the spinning time of the PS fiber. The PEN substrate was used to set the transmittance baseline. The maximum transmittances of the Ag fiber electrodes with 40 nm Ag thickness were 97.26%, 94.41%, 92.40%, and 88.91% at spinning times of 5, 15, 30, and 60 s, respectively. In contrast, the maximum transmittances of the Ag-fiber/graphene hybrid electrodes were 93.48%, 90.06%, 88.75%, and 87.75%, respectively, which are approximately 3% smaller than those of the pristine Ag fiber electrodes; this reduction is more than the well-known transmittance reduction of 2.3% achieved by the monolayer graphene, which may be attributed to impurities at the surface of the graphene layer. However, this result is considered reasonable as the transmittance difference was only approximately 3%. The sheet resistances of the Ag fiber electrodes obtained at spinning times of 5, 15, 30, and 60 s were 108.6, 45.7, 29.2, and 15.98 Ω/sq, respectively, as shown in Fig. 3c. After the introduction of graphene, the sheet resistances were 67.2, 30.9, 18.51, and 11.94 Ω/sq, respectively, which are approximately 38% lower in comparison with those of the Ag fiber electrodes.

For both Ag fiber electrodes and Ag-fiber/graphene hybrid electrodes, the transmittance of the substrate decreased as the spinning time of PS fiber increased owing to the increase in surface coverage with increase in the spinning time. The surface coverage of Ag fiber electrodes, as a function of spinning time, is discussed in our previous paper^30^. On the contrary, as the surface coverage increased, the network of the Ag fiber also increased, and the overall conductivity improved. Figure 3d shows the figure of merit (FoM), which represents the trade-off between the transmittance and sheet resistance of Ag-fiber/graphene hybrid electrodes as a function of spinning time. The transmittance at 550 nm decreased gradually as the spinning time increased, but the overall FoM increased as the resistance decreased.

Compared to those of the Ag fiber electrodes, the transmittance and sheet resistance of the Ag-fiber/graphene hybrid electrodes were higher and lower, respectively, which suggests that the Ag-fiber/graphene hybrid electrodes delivered better optical and electrical performances than the Ag fiber electrodes. This is because, as mentioned earlier, since the Ag fiber electrodes feature a network form, there are empty spaces where the electrodes are not present, which eventually leads to an open-circuit fault. In other words, the sheet resistance increased as the connection between the Ag fiber electrodes and the conductive probe was partially broken, and for the Ag-fiber/graphene hybrid electrodes, the CVD graphene layer covered the entire Ag fiber electrodes, which means that the current could flow through every space tightly, leading to a seamless physical contact with the conductive probe. Furthermore, the CVD graphene layer is known to be a highly stable and efficient electron transport layer. Thus, the current measurement between the Ag-fiber/graphene hybrid electrodes and the conductive probe could be highly effective, which means that the Ag-fiber/graphene hybrid electrodes are expected to provide stable electric signals and power supply. An improved electrical connection increases the potential of the Ag-fiber/graphene hybrid electrodes for application as an alternative to the existing electrodes for practical optoelectronic devices.

### Mechanical and chemical characteristics of the Ag-fiber/graphene hybrid electrodes

The Ag-fiber/graphene hybrid electrodes exhibited superior mechanical flexibilities, essential for emerging flexible optoelectronic devices such as OLEDs and OSCs. A repetitive bending test was performed to investigate their mechanical durability. During the test, pieces of the Ag-fiber/PEN and Ag-fiber/graphene/PEN were rounded at various bending radius (*r*_b_) or rolled around and subsequently unrolled under a 5 mm bending radius at a speed of 3 mm s^−1^. The sheet resistance of each electrode was compared to its initial value (Δ*R*/*R*_0_, where Δ*R* is the resistance change after the bending, and *R*_0_ is the initial resistance). The tensile strain applied to the bent substrate was estimated by^31^

$${\rm{Strain}}( \% )=\,\frac{{d}_{substrate}+{d}_{electrode}}{2\times {R}_{c}},$$

where ${d}_{substrate}$ and ${d}_{electrode}$ are the thicknesses of the substrate and electrodes, respectively, and ${R}_{c}$ is the radius of curvature.

**Figure 4:**
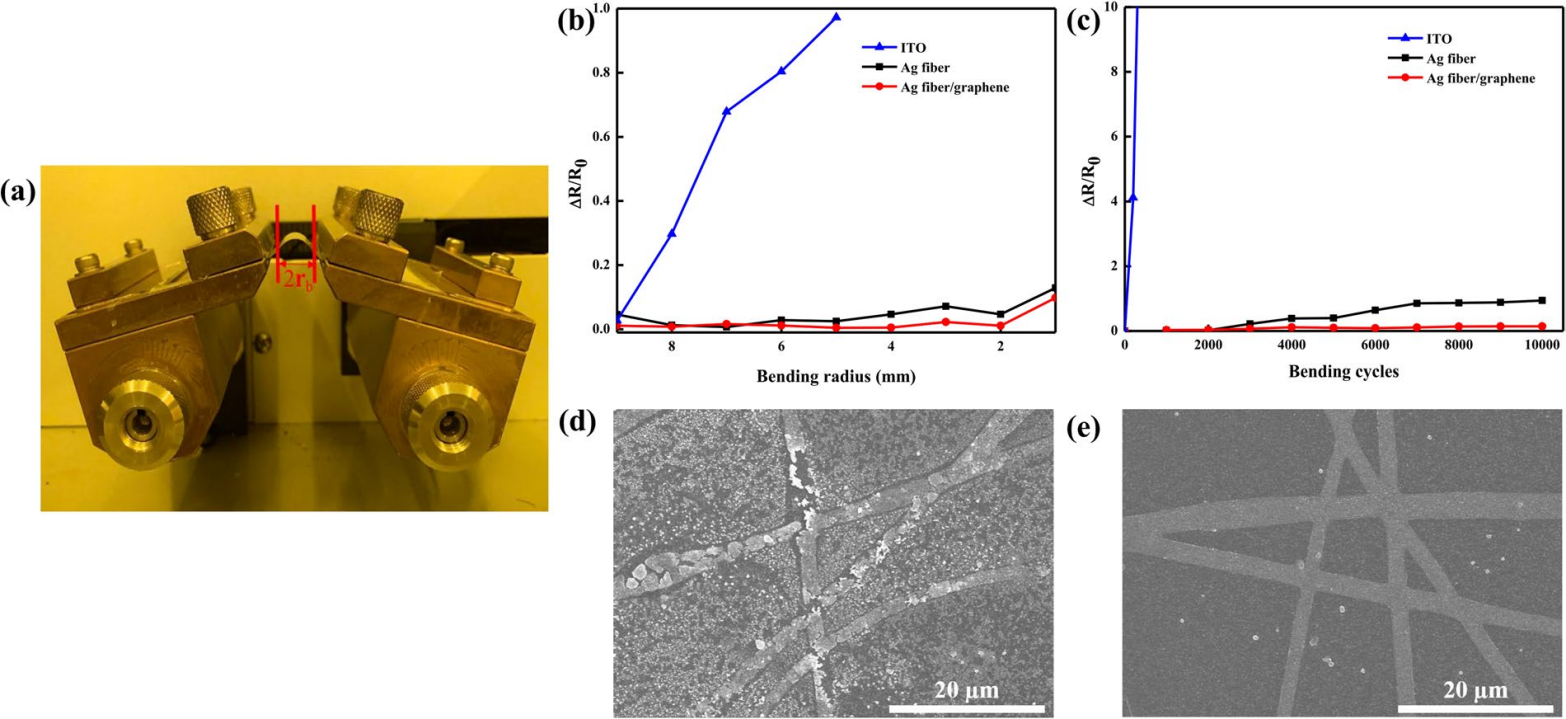
(**a**) Photographs of the bending tester, and hybrid electrodes rolled by the bending tester; Relative difference in resistances as a function of the (**b**) radius of curvature and (**c**) number of bending cycles at a bending radius of 5 mm; SEM images of the (**d**) Ag fiber and (**e**) Ag-fiber/graphene after the bending test.

In Fig. 4b, the mechanical flexibilities of ITO, Ag fiber, and Ag-fiber/graphene on PEN are compared at various bending radii (*r*_b_). It is well known that the ITO is made of brittle materials, so it cracks upon exposure to a minimal strain. Therefore, the resistance of the ITO film rapidly increased up to 100% above *r*_b_ = 5 mm. Both Ag fiber film and Ag-fiber/graphene film exhibited changes in resistance approximately smaller than 15% at difference *r*_b_ values, although the resistance change of the Ag fiber film was 5% larger than that of the Ag-fiber/graphene film. Figure 4c compares the mechanical flexibilities of the Ag fiber and Ag-fiber/graphene on the PEN as functions of the number of wrapping cycles. The resistance of the Ag-fiber/graphene hybrid film was slightly changed after 2,000 bending cycles, whereas that of the Ag fiber film was increased by more than 20% after the 2,000 bending cycles and by 100% after 10,000 bending cycles. As shown in Fig. 4d, after the 10,000 cycles of bending, cracks were initiated in the bent regions of the Ag fiber electrodes, which detached a part of the conductive layer and thus led to the increase in resistance. In contrast, because of the mechanical robustness of graphene, the Ag-fiber/graphene hybrid film was highly tolerant to bending and showed excellent mechanical stability. As shown in Fig. 4e, the Ag-fiber/graphene hybrid film did not exhibit cracks. Consequently, the monolayer graphene film enhanced the adhesion of the hybrid electrodes to the substrate and stabilized the Ag fiber electrodes.

**Figure 5:**
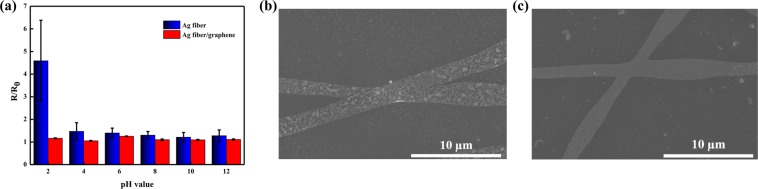
(**a**) Variations in the resistances of Ag fiber and Ag-fiber/graphene immersed in solutions with different pH values; SEM images of the (**b**) Ag fiber and (**c**) Ag-fiber/graphene after the immersion in the solution with a pH of 2.

To further evaluate whether the graphene layer acted as a chemically protective layer against the Ag fiber electrodes, various solutions with different pH values of 2 to 12 were prepared. The Ag fiber and Ag-fiber/graphene electrodes were then covered in each pH solution for 20 min, respectively. As shown in Fig. 5a, the resistance of the Ag fiber electrodes immersed in the solutions with a pH of 4 to 12 were not significantly different, whereas, at a pH of 2, the resistance change was approximately 4.5%. The resistance of the Ag-fiber/graphene hybrid electrodes was not considerably increased in the entire pH range of 2–12. The graphene’s unique honeycomb lattice acts as a protective barrier that prevents the penetration of small molecules. Therefore, the Ag fiber was able to maintain the properties of the electrode without oxidation, due to the characteristics of the graphene. The Ag-fiber/graphene hybrid electrodes exhibited superior chemical stability. Figure 4b shows that the morphology of the Ag-fiber/graphene hybrid electrodes was remarkably stable, whereas that of the pristine Ag fiber exhibited defects induced by corrosion in the chemical stability test.

### Thermal stability of the Ag-fiber/graphene hybrid electrodes

**Figure 6:**
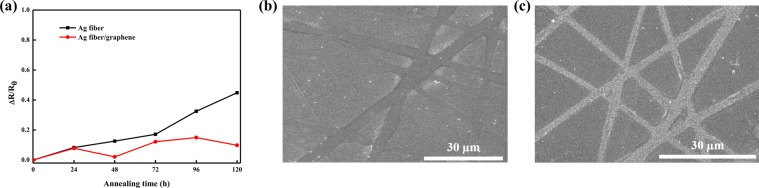
(**a**) Variations in the resistances of the Ag fiber and Ag-fiber/graphene when exposed to a temperature of 85 °C; SEM images of the (**b**) Ag fiber and (**c**) Ag-fiber/graphene after the thermal test.

To further evaluate the thermal stability of the Ag-fiber/graphene hybrid electrodes through the thermal-stable property of the graphene protection layer, the Ag fiber and Ag-fiber/graphene electrodes on the PEN substrates were annealed on a hot plate at 85 °C for 120 h. Their variations in resistance were measured after every 24 h. The melting point of the Ag fiber was significantly low (85 °C), even though the melting point of silver is approximately 962 °C, owing to the increased influence of the loosely bounded surface atoms of the nanostructure^32,33^. As shown in Fig. 6a, the resistance of the Ag fiber electrodes rapidly increased after 24 h and then reached a value that was almost 50% higher than the initial resistance. After the Ag fiber electrodes melted at 85 °C, the contact resistance decreased, and the percolation network was broken upon thermal treatment for 48 h^32^. It is well known that Ag electrodes, such as the Ag nanowires and the Ag fiber, quickly oxidize when exposed to air. While the Ag electrodes are oxidized, the silver oxides are formed on the Ag fiber electrodes surface, which leads to an increase in the electrical resistivity and junction resistance of the Ag fiber^34^, as shown in Fig. 6b. In contrast, the Ag-fiber/graphene hybrid electrodes exhibited a slightly (less than 10%) increased resistance, which demonstrates its superior thermal stability to that of the pristine Ag fiber electrodes. Although few Ag-fiber/graphene hybrid structures were rolled into balls, as shown in Fig. 6c, no electrode disconnections were observed. The significant gap in the change in sheet resistance between the Ag fiber and Ag-fiber/graphene hybrid electrodes verifies that graphene has high thermal stability in the atmosphere owing to its high heat conductivity. Furthermore, the graphene protective layer proves to act as an excellent gas-barrier layer for the underlying Ag fiber electrodes owing to its impermeability to small molecules such as oxygen.

The performances of Ag fiber electrodes and Ag-fiber/graphene hybrid electrodes are summarized in Table 2. The fabricated Ag-fiber/graphene hybrid electrodes exhibit excellent performance, in comparison with Ag fiber electrodes—62.2% lower sheet resistance with excellent mechanical, thermal, and chemical stabilities. The overall result suggests that Ag-fiber/graphene hybrid electrodes can improve the performance and lifespan of emerging optoelectronic devices.

**Table 2:** Comparison of Ag fiber electrodes and Ag-fiber/graphene hybrid electrodes.

Specifications	Ag fiber electrodes	Ag-fiber/graphene hybrid electrodes
Total transmittance (550 nm) [%]	90.17	87.57
Sheet resistance [Ω/sq]	31.3	19.46
Mechanical stability (5 mm for 10000 cycles) [ΔR/R_0_]	0.94	0.15
Thermal stability (85 °C for 120 h) [ΔR/R_0_]	0.45	0.1
Chemical stability (pH 2) [R/R_0_]	4.59	1.79

## Conclusions

The highly transparent, flexible and stable Ag-fiber/graphene hybrid electrodes were fabricated by wet-transferring the CVD graphene onto the Ag fiber electrodes. The Ag-fiber/graphene hybrid electrodes featured excellent optical and electrical properties, chemical stability, and mechanical flexibility, and they exhibited very low surface roughnesses, as the CVD graphene layer simultaneously served as an efficient current pathway by filling the voids. Furthermore, the Ag-fiber/graphene hybrid electrodes exhibited superior long-term stability under harsh environments, such as high temperature or chemical disturbances. Overall, the use of graphene as the buffer layer in the Ag fiber electrodes ensures high stretchability, low surface roughness, and high chemical and thermal stabilities while retaining the properties of the Ag fiber electrodes. The proposed unconventional approach can advance the development of transparent electrodes for emerging electronics.

## Methods

### Preparation of junction-free Ag fiber electrodes by electro-spinning

First, $2.5\,\text{cm}\,\times 2.5\,\text{cm}$ PEN substrate were ultraviolet-ozone (UV-O) treated for 15 min. Then, by the CVD method, Ag was deposited on the PEN substrates with thicknesses of 20, 40, 80, and 160 nm. To prepare the PS fiber, a PS solution (14 wt%) was obtained by dissolving 400 mg of PS (Sigma-Aldrich Korea Ltd.) in acetone and dimethylformamide (DMF). The electrospinning of the PS solution on the silver-deposited PEN substrates was then conducted, which yielded the PS fiber. In order to lead cross-linking, the electro-spun PS fiber was then heated on a hot plate at 130 °C for 10 min. The substrates were subjected to an oxygen (O_2_) plasma under a pressure of 500 mTorr at 90 W during 5 min to oxidize the Ag thin film not covered with the PS fiber mask. Then the substrate was immersed in an H_2_O_2_ solution to etch the oxidized Ag thin film (AgO_X_) and then immersed in deionized (DI) water for 30 s for complete removal of AgO_X_. Finally, it was left for 5 min in chloroform to remove the remaining PS fiber. The fabrication is also presented in detail in our previous report^30^.

### Fabrication of the Ag-fiber/graphene hybrid electrodes

The Ag-fiber/graphene hybrid electrodes were fabricated by wet-transferring CVD graphene layer on top of the Ag fiber electrodes. A graphene monolayer was transferred by using the poly (methyl methacrylate) (PMMA)-based technique (PMMA, MicroChem 950 C4, 4% in chlorobenzene, molecular weight: 950,000). A PMMA layer was spin-coated on a graphene/copper foil at 500 rpm for 15 s and 2,000 rpm for 30 s. The PMMA layer could act as a support layer for the graphene. After the coating, the sample was annealed at 100 °C for 60 s and dried in air. The sample was floated on an ammonium persulfate etchant (0.1 M in DI water) for 12 h until the copper foil was fully etched away. A PEN substrate with electro-spun Ag fiber electrodes was used to lift off the PMMA/graphene and then dried in air for 8 h to remove water residues trapped between the graphene and PET substrate. The sample was then immersed in acetone, isopropyl alcohol, and DI water (for 15 min in each of them) to remove the PMMA layer.

### Measurements

A four-probe system was used to measure the sheet resistance over randomly selected points of each sample to estimate the electrical properties of the Ag-fiber/graphene hybrid electrodes. The Ag-fiber/graphene hybrid electrodes were analysed by field-emission scanning electron microscopy (SEM; S-4800, Hitachi, Ltd.). Their optical properties were measured by using a Cary 5000 UV–visible spectrophotometer (Varian/Agilent). The surface topography and profiles of the hybrid electrodes were measured by using atomic force microscopy (AFM; XE-100, Park Systems Inc.). A bending tester (z-tec, Inc.) with a digital multimetre was used to perform a cyclic bending test. Raman spectroscopy (LabRam ARAMIS IR2, HORIBA Jobin Yvon) at a laser excitation wavelength of 532 nm was employed to measure the graphene quality. pH solutions were prepared by mixing a solution of hydrochloric acid (HCl) and sodium hydroxide (NaOH) with DI water to test the chemical stabilities.

## Data Availability

All data generated or analysed during this study are included in this published article.
